# What is new in the 2017 ESC clinical practice guidelines: Management of acute myocardial infarction in patients presenting with ST-segment elevation

**DOI:** 10.1007/s00508-018-1333-0

**Published:** 2018-05-23

**Authors:** Irene M. Lang

**Affiliations:** Department of Internal Medicine II, Division of Cardiology, Vienna General Hospital, Medical University of Vienna, Währinger Gürtel 18-20, 1090 Vienna, Austria

**Keywords:** Atherosclerosis, Heart disease, Acute coronary syndromes

## Abstract

Guidelines and recommendations are designed to guide physicians in making decisions in daily practice. Guidelines provide a condensed summary of all available evidence at the time of the writing process. Recommendations take into account the risk-benefit ratio of particular diagnostic or therapeutic means and the impact on outcome, but not monetary or political considerations. Guidelines are not substitutes but are complementary to textbooks and cover the European Society of Cardiology (ESC) core curriculum topics. The level of evidence and the strength of recommendations of particular treatment options were recently newly weighted and graded according to predefined scales. Guidelines endorsement and implementation strategies are based on abridged pocket guidelines versions, electronic version for digital applications, translations into the national languages or extracts with reference to main changes since the last version. The present article represents a condensed summary of new and practically relevant items contained in the 2017 European Society of Cardiology (ESC) guidelines for the management of acute myocardial infarction in patients with ST-segment elevation, with reference to key citations.

**Fig. 1:**
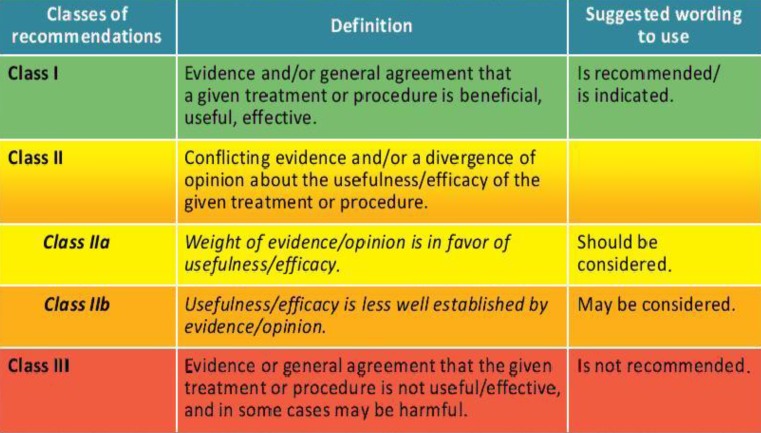
Classes of recommendations [1]. Reproduced by permission of Oxford University Press on behalf of the European Society of Cardiology. © The European Society of Cardiology 2017. All rights reserved. For permissions please email journals.permissions@oup.com. This figure is not included under the Creative Commons CC BY license of this publication. Please visit: www.escardio.org/Guidelines/

**Fig. 2:**
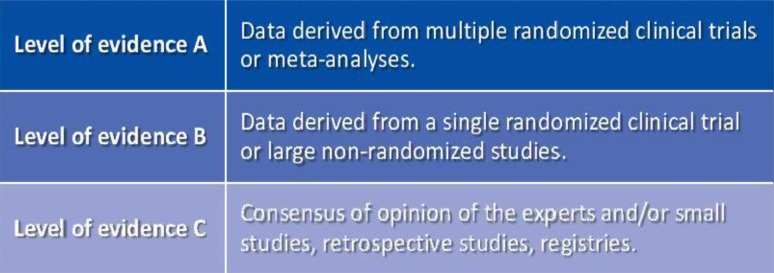
Level of evidence [1]. Reproduced by permission of Oxford University Press on behalf of the European Society of Cardiology. © The European Society of Cardiology 2017. All rights reserved. For permissions please email journals.permissions@oup.com. This figure is not included under the Creative Commons CC BY license of this publication. Please visit: www.escardio.org/Guidelines/

## Radial access is recommended over femoral access if performed by an experienced radial operator (1, A)

In a recent randomized, multicenter, superiority trial [2] of 8404 patients with acute coronary syndrome (ACS), radial angiography and percutaneous coronary intervention (PCI) reduced non-coronary artery bypass surgery (CABG)-associated bleeding (RR 0.67) and all-cause mortality (RR 0.72) compared with femoral access. The data reinforce previous observations from the radial versus femoral access for coronary intervention (RIVAL) access for coronary intervention trial [3], and the radial versus femoral randomized investigation in ST elevation acute coronary syndrome (RIFLE-STEACS) trial [4].

## Stenting with new generation drug-eluting stents (DES) is recommended over bare metal stents (BMS) for primary PCI (1, A)

In a multicenter, multinational, prospective, randomized, single-blinded, controlled trial in patients with ST elevation myocardial infarction (STEMI), the everolimus-eluting stents (EES) versus bare-metal stents (BMS) in ST-segment elevation myocardial infarction (EXAMINATION) trial, the combined endpoint of all-cause death, any recurrent myocardial infarction (MI), and any revascularization, target lesion revascularization and stent thrombosis occurred in 108 (14.4% of 751 patients of the EES group) and in 129 (17.3% of 747 patients of the BMS group, *p* = 0.11) at 2 years. The rates of target lesion revascularization and stent thrombosis were significantly lower in the EES group than in the BMS group. Thus, safety and efficacy of EES compared with BMS in the setting of STEMI were confirmed.

In another study [5], 9013 patients undergoing any PCI were randomized to implantation of EES, zotarolimus-eluting stents or BMS. At 6 years, there were no significant between-group differences in the composite of death from any cause and nonfatal spontaneous MI; however, the 6‑year rates of any repeat revascularization were 16.5% in the group receiving DES and 19.8% in the group receiving BMS (hazard ratio, 0.76; 95% confidence interval CI, 0.69–0.85; *P* < 0.001) and the rates of definite stent thrombosis were 0.8% and 1.2%, respectively (*P* = 0.0498).

In the comparison of biolimus eluted from an erodible stent coating with bare metal stents in acute ST elevation myocardial infarction (COMFORTABLE) trial [6] 1161 STEMI patients were randomly assigned to biolimus-eluting stent (BES) or BMS. At 2 years, differences in the primary end point of cardiac death, target vessel MI, and target lesion revascularization continued to diverge in favor of BES-treated patients (5.8%) compared with BMS-treated patients (11.9%; *P* < 0.001), with a significant risk reduction during the second year of follow-up (hazard ratio 1–2 years = 0.45; 95% CI, 0.20–1.00; *P* = 0.049). At 13 months, angiographic in-stent diameter stenosis was less in BES-treated lesions (12.0 ± 7.2%) than in BMS-treated lesions (39.6 ± 25.2%, *P* < 0.001).

## Routine revascularization of non-IRA lesions should be considered in STEMI patients with multivessel disease before hospital discharge (IIa, C)

In patients with STEMI and multivessel coronary artery disease undergoing infarct artery PCI, preventive PCI in non-infarct coronary arteries with major stenosis (*n* = 234) significantly reduced the risk of death from cardiac causes (0.34; 95% CI, 0.11–1.08), nonfatal MI (0.32; 95% CI, 0.13–0.75) or refractory angina (0.35; 95% CI, 0.18–0.69) compared with no preventive PCI (231 patients), with 9 events per 100 patients and 23 per 100, respectively [7].

In a prospective study [8], 313 patients were 1:1 randomized to no further invasive treatment after primary PCI (PPCI) of the infarct-related artery only and 314 were assigned to fractional flow reserve (FFR) guided complete revascularization. Events comprising the primary endpoint were recorded in 68 (22%) patients who had PCI of the infarct-related artery only and in 40 (13%) patients who had complete revascularization (hazard ratio 0.56, 95% CI 0.38–0.83; *p* = 0.004). The data show that complete revascularization guided by FFR measurements significantly reduces the risk of future events.

Similar data resulted from a further study that assigned 885 patients with STEMI and multivessel disease who had undergone PPCI of an infarct-related coronary artery in a 1:2 ratio to undergo complete FFR-guided revascularization of non-infarct-related coronary arteries (295 patients) or to not undergo revascularization of non-infarct-related coronary arteries (590 patients) [9] and 8 versus 21 events occurred per 100 patients, respectively.

The complete versus lesion-only primary PCI trial (CvLPRIT) was a UK open-label randomized study comparing complete revascularization at index admission with treatment of the infarct-related artery (IRA) only [10] and randomized 296 patients in 7 UK centers. Complete revascularization was performed either at the time of PPCI or before hospital discharge (*n* = 150). A composite of all-cause death, recurrent MI, heart failure, and ischemia-driven revascularization within 12 months occurred in 10.0% of the complete revascularization group versus 21.2% in the IRA only revascularization group (*n* = 146).

## In patients with heparin-induced thrombocytopenia, bivalirudin is recommended as the anticoagulant agent during primary PCI (I, C); however, the recommendation to routinely use bivalirudin has been weakened (was 1, A, is now IIa, A)

The MATRIX trial including 8404 patients with acute coronary syndrome, with or without ST-segment elevation, demonstrated that the use of radial access compared with femoral access decreased net adverse clinical events. In a posthoc analysis of 7213 patients who were randomly allocated to bivalirudin or unfractionated heparin, no evidence was found for an interaction between the effect of radial versus femoral access and allocation to bivalirudin or unfractionated heparin for the two co-primary outcomes, all-cause mortality, or Bleeding Academic Research Consortium (BARC) 3 or 5 bleeding (*p* for interaction ≥0.64), although bivalirudin was used during percutaneous coronary intervention in more than 40% of patients [2].

In an open-label, randomized controlled trial, 1812 patients undergoing PPCI at Liverpool Heart and Chest Hospital [11] were randomly allocated (1:1) to heparin (70 U/kg body weight) or bivalirudin (bolus 0.75 mg/kg; infusion 1.75 mg/kg per h). The primary efficacy outcome occurred in 79 (8.7%) of 905 patients in the bivalirudin group and 52 (5.7%) of 907 patients in the heparin group (absolute risk difference 3.0%; relative risk RR 1.52, 95% CI 1.09–2.13, *p* = 0.01). The primary safety outcome occurred in 32 (3.5%) of 905 patients in the bivalirudin group and 28 (3.1%) of 907 patients in the heparin group (absolute risk difference 0.4%; relative risk [RR] 1.15, 95% CI 0.70–1.89, *p* = 0.59). The data show that compared with bivalirudin, heparin reduces the incidence of major adverse ischemic events in the setting of PPCI, with no increase in bleeding complications.

## Routine use of i. v. enoxaparin should be considered in STE-ACS in the context of primary PCI (IIa, A)

An i. v. bolus of enoxaparin 0.5 mg/kg was compared with UFH in the randomized open-label Acute MI Treated with primary angioplasty and inTravenous enOxaparin or unfractionated heparin to Lower ischemic and bleeding events at short and Long-term follow-up (ATOLL) trial, including 910 STEMI patients [12]. The primary composite endpoint of 30-day death, MI, procedural failure, or major bleeding was not significantly reduced by enoxaparin (17% relative risk reduction, *P* = 0.063), but there was a reduction in the composite main secondary endpoint of death, recurrent MI or ACS, or urgent revascularization. Importantly, there was no evidence of increased bleeding with enoxaparin [13].

In a meta-analysis of 23 PCI trials (30,966 patients, 33% PPCI), enoxaparin was associated with a significant reduction in death compared to UHF. This effect was particularly significant in the context of PPCI and was associated with a reduction in major bleeding [14].

## Cangrelor may be considered in the catheter laboratory if P2Y12 inhibitors have not been given (IIB, A)

A prespecified, pooled analysis of patient-level data from three trials (CHAMPION-PCI, CHAMPION-PLATFORM, and CHAMPION-PHOENIX) compared cangrelor with control (clopidogrel or placebo) for prevention of thrombotic complications during and after PCI. Trial participants were patients undergoing PPCI for STEMI (11.6%), non-ST-elevation ACS (57.4%), and PCI for stable coronary artery disease (31.0%). Efficacy was assessed in the modified intention-to-treat population of 24,910 patients. Cangrelor reduced the odds of death, MI, ischemia-driven revascularization, or stent thrombosis at 48 h by 19% (*p* = 0.0007), and stent thrombosis by 41% (*p* = 0.0008); however, cangrelor increased global use of strategies to open occluded coronary arteries (GUSTO) mild bleeding (16.8% vs. 13.0%, *p* < 0.0001) [15].

## Routine use of thrombus aspiration is not recommended (III, A)

The TOTAL trial assigned 10,732 patients with STEMI undergoing PPCI to a strategy of routine upfront manual thrombectomy versus PCI alone. Routine manual thrombectomy did not reduce the risk of cardiovascular death, recurrent MI, cardiogenic shock, or NYHA class IV heart failure within 180 days but was associated with an increased rate of stroke within 30 days (hazard ratio, 2.06; 95% CI, 1.13–3.75; *P* = 0.02) [16].

The TASTE trial was a multicenter, prospective, randomized, controlled, open-label clinical trial, with enrolment of patients from the national comprehensive Swedish coronary angiography and angioplasty registry (SCAAR). A total of 7244 patients with STEMI undergoing PCI were randomly assigned to manual thrombus aspiration followed by PCI or to PCI only. Routine thrombus aspiration before PCI did not reduce 30-day mortality. There were no significant differences between the groups with respect to the rate of stroke or neurologic complications at the time of discharge (*P* = 0.87) [17].

## Early discharge (within 48–72 h) should be considered appropriate in selected low-risk patients if early rehabilitation and adequate follow-up are arranged (IIa, A)

The optimal length of stay in the CCU/ICCU and hospital should be determined on an individual basis. Data are accumulating that after appropriate risk stratification, early discharge is safe [18–22].

## Routine oxygen is not recommended in patients with SaO2 ≥ 90% (III, B)

The DETO2X-AMI trial randomly assigned 6629 patients with suspected MI and an oxygen saturation of 90% or higher to receive either supplemental oxygen (6 l per min for 6–12 h, delivered through an open face mask) or ambient air. The median duration of oxygen therapy was 11.6 h, and the median oxygen saturation at the end of treatment was 99% among patients assigned to oxygen and 97% among patients assigned to ambient air. Hypoxemia developed in 62 patients (1.9%) in the oxygen group, compared with 254 patients (7.7%) in the ambient air group. The primary end point of death from any cause within 1 year after randomization occurred in 5.0% of patients assigned to oxygen and in 5.1% of patients assigned to ambient air (*P* = 0.80) [23].

## If indicated a 50% dose of tenecteplase should be considered in patients ≥75 years old (IIa, B)

The STREAM trial randomly assigned 1892 patients with STEMI who presented within 3 h after symptom onset and who were unable to undergo PPCI within 1 h to PPCI or fibrinolytic therapy with bolus tenecteplase, clopidogrel, and enoxaparin. A composite of death, shock, congestive heart failure, or reinfarction up to 30 days occurred in 116 of the 939 patients (12.4%) in the fibrinolysis group and in 135 out of 943 patients (14.3%) in the PPCI group (*P* = 0.21). More intracranial hemorrhages occurred in the fibrinolysis group than in the PPCI group (1.0% vs. 0.2%, *P* = 0.04; after protocol amendment with a 50% dose reduction of tenecteplase, 0.5% vs. 0.3%, *P* = 0.45) [24].

## In patients with LDL-C > 1.8 mmol/l (>70 mg/dl) despite a maximum tolerated statin dose who remain at high risk, further therapy to reduce LDL-C should be considered (IIa, A)

A meta-analysis of trials comparing more intensive against less intensive lowering of low-density lipoprotein cholesterol (LDL-C) with statins indicated that more intensive statin therapy produced greater reductions in the risks of cardiovascular death, non-fatal MI, ischemic stroke, and coronary revascularization [25]. For every mmol/l reduction in LDL-C, these further reductions in risk were similar to the proportional reductions in the trials of statins vs. control.

## In high ischemic-risk patients who have tolerated DAPT without a bleeding complication treatment with DAPT in the form of ticagrelor 60 mg twice a day on top of low-dose aspirin may be considered for up to 3 years (IIb, B)

A total of 21,162 patients after recent MI were randomly assigned to ticagrelor at a dose of 90 mg twice daily, ticagrelor at a dose of 60 mg twice daily or placebo. Patients were also given low-dose aspirin. The two ticagrelor doses each reduced the rate of the composite of cardiovascular death, MI, or stroke as compared with placebo. Rates of thrombolysis in myocardial infarction (TIMI) major bleeding were higher with ticagrelor (2.60% with 90 mg and 2.30% with 60 mg) than with placebo (1.06%, *P* < 0.001 for each dose vs. placebo).
